# CONFIRM: a double-blind, placebo-controlled phase III clinical trial investigating the effect of nivolumab in patients with relapsed mesothelioma: study protocol for a randomised controlled trial

**DOI:** 10.1186/s13063-018-2602-y

**Published:** 2018-04-18

**Authors:** Dean A. Fennell, Emma Kirkpatrick, Kelly Cozens, Mavis Nye, Jason Lester, Gerard Hanna, Nicola Steele, Peter Szlosarek, Sarah Danson, Joanne Lord, Christian Ottensmeier, Daniel Barnes, Stephanie Hill, Mihalis Kalevras, Tom Maishman, Gareth Griffiths

**Affiliations:** 1 University of Leicester and University Hospitals of Leicester NHS Trust, Leicester, UK; 2 Southampton Clinical Trials Unit, Centre for Cancer Immunology, University of Southampton, Southampton, UK; 3 Velindre NHS Trust, Cardiff, UK; 4 Queen’s University Belfast, Belfast, UK; 5 Beatson West of Scotland Cancer Centre, Glasgow, UK; 6 Queen Mary University of London, London, UK; 7 Academic Unit of Clinical Oncology, Weston Park Hospital, University of Sheffield, Sheffield, UK; 8 Southampton Health Technology Assessment Centre, University of Southampton, Southampton, UK; 9 Cancer Sciences Unit, Faculty of Medicine, University of Southampton and Experimental Cancer Medicine Centre, Southampton, UK

**Keywords:** Mesothelioma, PD-L1, Anti-PD-1, Nivolumab, Randomised controlled trial, Quality of life, Overall survival, Placebo, Intention to treat

## Abstract

**Background:**

Mesothelioma is an incurable, apoptosis-resistant cancer caused in most cases by previous exposure to asbestos and is increasing in incidence. It represents a growing health burden but remains under-researched, with limited treatment options. Early promising signals of activity relating to both PD-L1- and PD-1-targeted treatment in mesothelioma implicate a dependency of mesothelioma on this immune checkpoint. There is a need to evaluate checkpoint inhibitors in patients with relapsed mesothelioma where treatment options are limited.

**Methods:**

The addition of 12 months of nivolumab (anti-PD1 antibody) to standard practice will be conducted in the UK using a randomised, placebo-controlled phase III trial (the Cancer Research UK CONFIRM trial). A total of 336 patients with pleural or peritoneal mesothelioma who have received at least two prior lines of therapy will be recruited from UK secondary care sites. Patients will be randomised 2:1 (nivolumab:placebo), stratified according to epithelioid/non-epithelioid, to receive either 240 mg nivolumab monotherapy or saline placebo as a 30-min intravenous infusion. Treatment will be for up to 12 months. We will determine whether the use of nivolumab increases overall survival (the primary efficacy endpoint). Secondary endpoints will include progression-free survival, objective response rate, toxicity, quality of life and cost-effectiveness. Analysis will be performed according to the intention-to-treat principle using a Cox regression analysis for the primary endpoint (and for other time-to-event endpoints).

**Discussion:**

The outcome of this trial will provide evidence of the potential benefit of the use of nivolumab in the treatment of relapsed mesothelioma. If found to be clinically effective, safe and cost-effective it is likely to become the new standard of care in the UK.

**Trial registration:**

EudraCT Number: 2016–003111-35 (entered on 21 July 2016); ClinicalTrials.gov, ID: NCT03063450. Registered on 24 February 2017.

**Electronic supplementary material:**

The online version of this article (10.1186/s13063-018-2602-y) contains supplementary material, which is available to authorized users.

## Background

Mesothelioma is an incurable, apoptosis-resistant cancer caused in most cases by previous exposure to asbestos and is increasing in incidence in the UK and beyond [1, 2]. The majority of patients with mesothelioma present with advanced disease and prognosis is poor, especially with sarcomatoid mesothelioma. Mesothelioma therefore represents a growing health burden, but it remains under-researched and treatment options are limited. Chemotherapy is currently the standard of care in the first-line setting in which two positive, randomised phase III trials have been reported, showing improved survival with the addition of pemetrexed or raltitrexed to cisplatin, respectively [3, 4]. The recent French MAPS trial has shown that the addition of bevacizumab to pemetrexed-cisplatin and bevacizumab maintenance, improves survival from 16.1 months within the control arm to 18.8 months with the addition of bevacizumab [5].

Unfortunately, even following successful first-line therapy, all patients with mesothelioma will subsequently relapse. There is currently no standard second-line therapy; however, it is common practice to re-challenge with the first-line regimen, usually pemetrexed-cisplatin, if there has been a reasonable progression-free interval. In addition to this, vinorelbine is used in some centres, as phase II trials have shown this drug to have promising activity in the second-line treatment of mesothelioma. Currently the Vinorelbine In Mesothelioma study (VIM study: NCT02139904) trial is ongoing to evaluate its efficacy in this setting. Due to the availability of second-line options, either within the VIM trial or off study, the CONFIRM trial aims to evaluate immunotherapy in the third-line setting, a clinical situation in which current standard of care is active symptom control only. Thus, best supportive care has been chosen as the comparator arm in this study.

The landscape of cancer therapy has been recently transformed by the emergence of immunotherapy involving the targeting of immune checkpoints [6–8]. Programmed cell death 1 (PD-1) is a 55-kDa transmembrane inhibitory immunoreceptor expressed by activated T cells that negatively regulates immune responses required for peripheral self-tolerance. PD-1 interacts with its ligand PD-L1, a member of the *B7* gene family, which is expressed on mesothelioma cells [9, 10]. The expression of PD-L1 (> 5% positively stained cells) has been reported in 40% of mesothelioma overall, with a higher rate in sarcomatoid mesotheliomas and is a poor prognostic factor. The PD-1-PD-L1 axis mediates an inhibitory signal to T cells leading to induction of apoptosis via PD-1 activation. Accordingly, PD-1 or PD-L1 blockade de-represses T-cell activation, unleashing a clinical immune response with tumour regression [11].

Targeting the PD-1 in mesothelioma has demonstrated promising efficacy. Of 25 patients receiving pembrolizumab in a single-arm phase I/II study (Keynote 28 (KN028)), the objective response rate was 20% in patients with PD-L1-positive malignant pleural mesothelioma (≥ 1% PD-L1-positive tumour cells by immunohistochemistry). Additionally, 52% of patients had stable disease, resulting in a disease control rate of 72% [12].

PD-L1 blockade has also demonstrated promising efficacy in patients with mesothelioma [13]. In a phase IB study (NCT01772004), 53 patients were treated with avelumab (MSB0010718C, Merck Serono), with histologically or cytologically confirmed unresectable mesothelioma (pleural or peritoneal) that progressed after a prior platinum-pemetrexed-containing regimen or a platinum-based regimen followed by pemetrexed. Avelumab was administered at a dose of 10 mg/kg as a 1-h infusion every two weeks (q2w) until confirmed progression, unacceptable toxicity, or any criteria for withdrawal occurred. Patients had received a median of 1.5 prior treatments (range, 0–7.4). Histology was epithelial (81.1%), mixed (11.3%) or sarcomatoid (3.8%). Objective responses were observed in 5 (9.4%) patients; all were partial responses (PR) and durable. Stable disease (SD) was observed in 9 additional patients (45%). The overall disease control rate (PR plus SD) was 56.6% (30 patients). Median progression-free survival (PFS) by Response Evaluation Criteria in Solid Tumours (RECIST) was 17.1 weeks (95% CI 6.1–30.1), and the PFS rate at 24 weeks was 38.4% (95% CI 23.3, 53.4).

Nivolumab is a human IgG4 anti-PD1 monoclonal antibody which blocks the PD-1 receptor on activated T cells, which has been approved by the Food and Drug Administration for the treatment of patients with unresectable or metastatic melanoma unresponsive to other drugs, and relapsed non-small-cell lung cancer, recurrent renal cancer and lymphoma. In a phase IIA clinical trial of nivolumab (3 mg/kg every two weeks (q2w) conducted at NKI, Amsterdam, the disease control rate at 12 weeks was 50% (*n* = 34) [14].

In summary, there is a need to find effective, safe, cost-effective interventions for individuals with mesothelioma. Using a two-arm, parallel-group randomised phase III trial (CONFIRM trial), we will compare nivolumab with placebo in patients with relapsed mesothelioma.

## Methods/design

This study protocol was written in accordance with Standard Protocol Items: Recommendations for Interventional Trials (SPIRIT). A SPIRIT checklist is provided in Additional file 1.

### Objectives

The main aim of the CONFIRM trial is to evaluate the efficacy, safety and cost-effectiveness of treatment with nivolumab in patients with relapsed mesothelioma.

### Study design

**Fig. 1:**
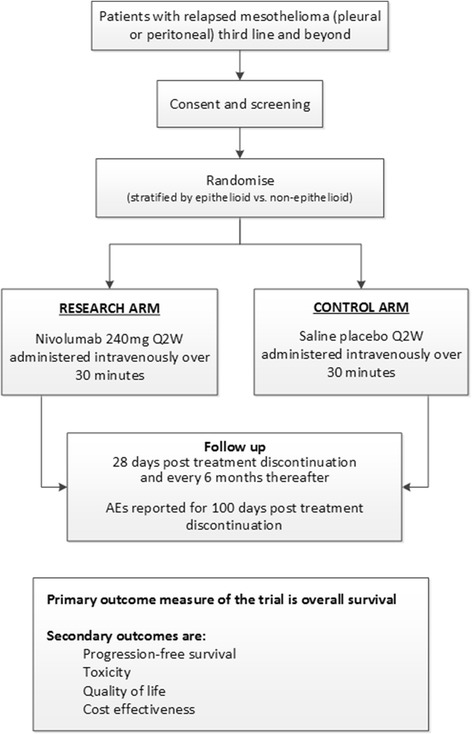
Trial schema

CONFIRM is a double-blind, placebo-controlled randomised phase III trial comparing nivolumab monotherapy versus placebo until disease progression, for a maximum of 12 months. Patients will be randomised by pharmacy staff at site using an Interactive Web Response System (IWRS) which will allocate participants in a 2:1 ratio to either the nivolumab or the control arm using the method of permuted blocks (NB. All investigators are blinded to the block size and stratification factors used until the end of the trial); see Fig. 1. Patients, clinicians and trial management staff will be blinded to treatment allocation. Treatment allocation will be unblinded only if there is a clinical reason that will affect decisions about how to proceed with patient care.

CONFIRM is being run in approximately 25 UK centres with the aim of recruiting a total of 336 patients.

CONFIRM has received ethical approval by the West Midlands – Edgbaston Research Ethics Committee (16/WM/0472) and has approval from the UK Medicines and Health Care Product Regulatory Agency (MHRA) to be conducted in the UK. Southampton Clinical Trials Unit (SCTU), a Cancer Research UK core-funded and UK Clinical Research Collaboration-registered CTU, is coordinating the trial. The University of Southampton is the sponsor for the trial https://www.southampton.ac.uk/research/ris.page. An independent Trial Steering Committee and Independent Data Monitoring Committee comprising two clinicians and a statistician experienced in this research area has been set up to monitor trial progress and safety. The CONFIRM Trial Management Group includes representatives from medical and clinical oncology, radiology, health economics and CTU staff involved in the day-to-day running of the trial. Charters for these groups are available via confirmtrial@soton.ac.uk.

The SCTU has undertaken a risk assessment for the CONFIRM trial which includes the requirements for monitoring (both central and site). The SCTU undertakes a number of internal audits of its own systems and processes annually and has routine audits from both its sponsor and the independent MHRA every 2–3 years.

### Primary outcome measure

The primary efficacy endpoint will be overall survival (OS – time to event). OS will be calculated as the time from randomisation to death from any cause. Those still alive will be censored at the time last known to be alive. NB. In addition to receiving participant data direct from treating hospitals we have also registered participants with England’s NHS Digital, or an equivalent in Wales, Scotland and Northern Ireland, to obtain primary outcome survival data.

### Secondary outcome measure

The secondary outcome measures are as follows:

- OS according to PD-L1 status
- Progression-free survival (PFS – time to event). PFS will be calculated from the date of randomisation to the date of disease progression (using modified RECIST or RECIST 1.1) or any death (whichever event comes first). Those still alive and progression free will be censored at the last time seen. Regular computerised tomography (CT) scans will not be mandated. Follow-up imaging will be as per local hospital policy and as clinically indicated
- Objective response rate (ORR) – assessed using modified RECIST or RECIST 1.1 during treatment and post treatment
- Quality of life (QoL) – assessed using EQ-5D-5L at baseline, after treatment cycles 3 and 6 and then at 28 days, 6 months and 1 year post treatment discontinuation. The EQ-5D-5 L assesses five dimensions: mobility, self-care, usual activities, pain/discomfort and anxiety/depression
- Toxicity – assessed using Common Terminology Criteria for Adverse Events (CTCAE) v4.03 at baseline, after each treatment cycle and at each follow-up visit
- Treatment compliance – assessed using treatment compliance Electronic Case Report Forms (eCRFs) during the treatment period
- Cost-effectiveness – assessed using plus EQ-5D-5 L and data on health resource usage during treatment and post discontinuation to calculate a cost per quality-adjusted life year (QALY)

### Sample size

The study aims to demonstrate that nivolumab will increase median overall survival (OS) of patients with relapsed mesothelioma from 6 months (estimated median OS [15]) to 8.5 months, equivalent to increasing the 6-month OS rate from 50% to 61.5%. To detect a hazard ratio (HR) of 0.70 with 80% power at a 4% two-sided significance level requires a total of 291 events (deaths). Recruiting 336 patients – randomised 2:1 (221 receiving nivolumab: 112 receiving placebo) – over a 4-year period and 6 months’ follow-up, should be sufficient to achieve this number of events.

It is anticipated that 40% of patients will be PD-L1 checkpoint positive [16]. This will allow us to have 80% power to detect a HR of 0.5 (*p* = 0.01) in OS, equivalent to increasing the 6 months OS rate from 50% to 70.7%. The number of patients required for this subgroup is 132 (88 nivolumab: 44 placebo) to achieve a total of 105 events (deaths).

Another important subgroup analysis to assess at the end of the trial is the OS of patients with a high PD-L1 expression of ≥ 50% (25% of patients are anticipated to have a high PD-L1 expression of ≥ 50%). The sample size will allow us to have 80% power in this subgroup to detect a HR of 0.4 (*p* = 0.01) in OS, equivalent to increasing the 6 months’ OS rate from 50% to 75.8% (i.e. number of patients in this subgroup is 78 (52 receiving nivolumab; 26 receiving placebo) with 59 events (deaths).

The trial is registered on the UK NIHR trial portfolio meaning that there are research nurses based at UK cancer hospitals who help in screening potential patients to identify those eligible for the trial.

### Study participants

#### Inclusion criteria

Participants should fulfil all the following criteria:

- Histological confirmation of mesothelioma (any subtype, pleural or peritoneal)
- Male or female ≥ 18 years old
- Patients must have received at least two prior lines of treatment (including patients who have had re-challenge with platinum/pemetrexed). Prior maintenance therapy is permitted but will not count as a line of treatment
- Prior lines of antineoplastic therapy, including chemotherapy, surgical resection of lesions, radiation therapy, must be completed at least 14 days prior to receiving study treatment
- Eastern Cooperative Oncology Group Performance Status (ECOG PS) 0–1
- Radiologically assessable disease by modified RECIST (pleural mesothelioma) or RECIST 1.1 (non-pleural mesothelioma or where measurements for mRECIST cannot be obtained). Radiological tumour assessment (CT scan) must be performed within 28 days of first dose of study treatment
- Evidence of disease progression by CT scan
- Prior palliative radiotherapy must have been completed at least 14 days prior to study drug administration
- Patients must be willing and able to comply with scheduled visits, treatment schedule, laboratory tests and other requirements of the study
- Screening laboratory values must meet the following criteria within 48 h prior to commencement of treatment: white blood cells ≥ 2 × 10^9^/L; neutrophils ≥ 1.5 × 10^9^/L; platelets ≥ 100 × 10^9^/L; haemoglobin ≥ 90 g/L; serum creatinine ≤ 1.5 × ULN or creatinine clearance (CrCl) > 50 mL/min (using the Cockcroft/Gault formula); female CrCl = [(140 − age in years) × weight in kg × 0.85) ÷ (72 × serum creatinine in μmol/L)]; male CrCl = [(140 − age in years) × weight in kg × 1.00) ÷ (72 × serum creatinine in μmol/L)]; aspartate aminotransferase (AST) ≤ 3 × ULN; alanine aminotransferase (ALT) ≤ 3 × ULN; total bilirubin ≤ 1.5 × ULN (except patients with Gilbert syndrome, who must have total bilirubin < 51.3 μmol/L)
- Expected survival of at least 12 weeks
- Appropriate contraception, negative pregnancy tests if of child-bearing potential and not breastfeeding
- Written informed consent, including use of tissue and blood samples for research

#### Exclusion criteria

Individuals meeting any of the following criteria will be excluded:

- Patients with untreated, symptomatic central nervous system (CNS) metastases, carcinomatous meningitis or active, known or suspected autoimmune disease. Participants are eligible if central nervous system (CNS) metastases are adequately treated and participants are neurologically returned to baseline (except for residual signs or symptoms related to the CNS treatment) for at least 2 weeks prior to treatment assignment. Participants must be either off corticosteroids, or on a stable or decreasing dose of less than or equal to 10 mg daily (or equivalent) for at least 2 weeks prior to treatment
- Patients with a condition requiring systemic treatment with either corticosteroids (> 10 mg daily prednisone equivalent) or other immunosuppressive medications within 14 days of the first dose of study drug administration
- Patients with active malignancy requiring concurrent intervention or previous malignancies (except non-melanoma skin cancers, and the following in-situ cancers: bladder, gastric, colon, endometrial, cervical/dysplasia, melanoma or breast) unless a complete remission was achieved at least 2 years prior to study entry *and* no additional therapy is required during the study period
- Any serious or uncontrolled medical disorder or active infection that, in the opinion of the investigator, may increase the risk associated with study participation, study drug administration, or would impair the ability of the patient to receive protocol therapy
- All toxicities attributed to prior anti-cancer therapy, other than alopecia and fatigue, not resolved to grade 1 (NCI CTCAE version 4.03) or baseline before administration of study drug
- Patients who have not recovered from the effects of major surgery or significant traumatic injury at least 14 days before the first dose of study treatment
- Known alcohol or drug abuse
- Patients who have received prior therapy with anti-PD-1, anti-PD-L1, anti-PD-L2, anti-CD137 or anti-CTLA-4 antibody (including ipilimumab or any other antibody or drug specifically targeting T-cell co-stimulation or checkpoint pathways) or who have previously taken part in a randomised Bristol Myers Squibb (BMS) clinical trial for nivolumab or ipilimumab including study CA209–743 (CheckMate 172)
- Testing positive for human immunodeficiency virus or known acquired immunodeficiency syndrome, or hepatitis B virus or hepatitis C virus indicating acute or chronic infection
- History of severe hypersensitivity reactions to other monoclonal antibodies

#### Withdrawal criteria

The participant/legal representative is free to withdraw consent from the study at any time without providing a reason. A participant could also withdraw from treatment but allow continuation of collection of data.

### Study procedure

#### Recruitment and consent

Patients are approached within a hospital setting and screened for eligibility by research staff to ensure that all inclusion and exclusion criteria are met. Informed consent to enter the trial is obtained from a patient by a clinician only after a full explanation has been given, an information leaflet offered and time allowed for consideration. Patients consent to provision of tumour and blood samples for use in laboratory studies including genetic analysis and for their data to be shared anonymously to support other research in the future (see Additional file 2). A list of study sites is available on request from confirmtrial@soton.ac.uk.

#### Baseline visit

Following informed consent, a CT scan with modified RECIST (pleural mesothelioma) or RECIST 1.1 (non-pleural mesothelioma) will be undertaken within 28 days of treatment. Participants will undergo physical examination including vital signs, oxygen saturation, measurement of height, weight, oxygen saturation and ECOG PS. Concomitant medications and medical history, including smoking history, exposure to asbestos and chronic pulmonary and cardiovascular diseases will be recorded. Safety assessments comprising full blood count, serum chemistry tests, liver and thyroid function tests will all be performed before randomisation. In addition, women of child-bearing potential will undertake a pregnancy test. Participants will be randomised within 48 h prior to commencement of treatment. Following randomisation participants will complete the European Quality of Life-5 Dimensions-5 Levels (EQ-5D-5 L) questionnaire and a formalin-fixed paraffin-embedded tumour-tissue block will be obtained, either archival or fresh if no archival sample is available.

#### Follow-up visits

**Fig. 2:**
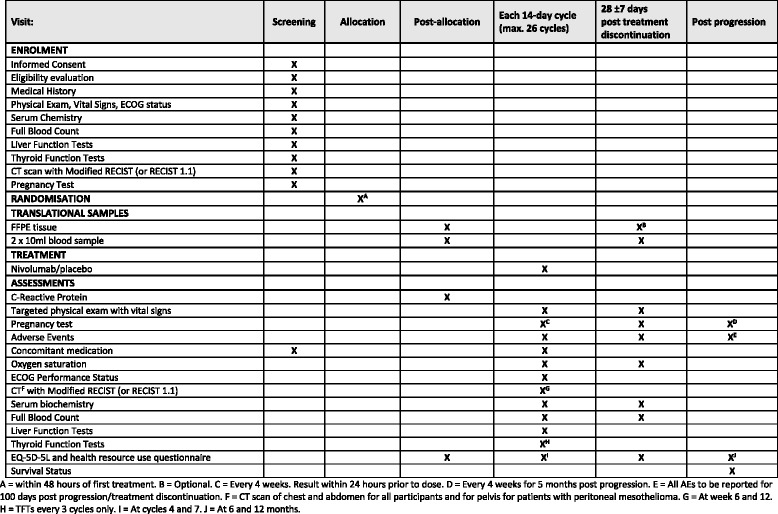
Schedule of procedures

Participants will attend hospital appointments for treatment every 14 days during treatment, 28 days post progression/treatment discontinuation, 6 months and 12 months (see Fig. 2). The follow-ups during the treatment period (i.e. until progression) will collect data required for the primary and secondary endpoints including disease assessments (NB. A CT scan will be carried out of chest and abdomen for all participants and for pelvis for patients with peritoneal mesothelioma), standard physical examinations, pregnancy tests (if appropriate), treatment compliance, ECOG PS, laboratory tests (e.g. urea, electrolytes, liver function, oxygen saturation, serum biochemistry, full blood count, lactate dehydrogenase (LDH) and C-reactive protein (CRP)) and adverse events. The participant will then enter a post-treatment/progression follow-up to collect data on adverse events, quality of life (QoL), health resource use and survival status. Serious adverse event (SAE) reporting is in real time to the SCTU safety desk throughout the study. SAEs are assessed to determine if related to drug treatment and whether unexpected or not, and subsequently reported to both BMS and the UK regulatory bodies.

### Data collection

Research staff at hospitals will complete trial eCRFs via a remote data collection tool (Medidata Rave). Data will be checked for missing or unusual values and checked for consistency within participants over time by SCTU trial staff. Any suspect data will be raised as data queries. Site staff will respond to the queries providing an explanation/resolution of the discrepancies. Full details on data management procedures are available in the Data Management Plan, available on request.

### Intervention

#### Nivolumab

Participants will receive nivolumab at a dose of 240 mg as a 30-min intravenous (IV) infusion, on day 1 ± 2 of every 14-day treatment cycle, until progression, unacceptable toxicity, withdrawal of consent, or the maximum treatment duration of 12 months is reached, whichever occurs first. There will be no dose escalations or reductions allowed. Patients may be dosed no less than 12 days from the previous dose.

The infusion must be administered through a sterile, non-pyrogenic, low-protein-binding, in-line filtre with a pore size of 0.2–1.2 μm. It should not be administered as an IV push or bolus injection. The total dose can be infused directly as a 10 mg/mL solution or can be diluted to as low as 1 mg/mL with sodium chloride 9 mg/mL (0.9%) solution for injection or glucose 50 mg/mL (5%) solution for injection. After administration the line should be flushed with sodium chloride 9 mg/ml (0.9%) solution for injection or 50 mg/ml (5%) glucose solution for injection. Participants should be carefully monitored for infusion reactions during administration.

#### Control

Patients who are randomised to receive placebo will receive sterile 0.9% sodium chloride as a 30-min IV infusion (as per the nivolumab treatment process).

Prohibited and restricted therapies during the trial (unless utilised to treat a drug-related adverse event) include immunosuppressive agents, any concurrent anti-neoplastic therapy and live vaccinations. Caution must be used with ototoxic or nephrotoxic concomitant drugs and discontinuation of the use of herbal medications prior to study enrolment is encouraged. Inhaled or topically administered steroids, and adrenal replacement steroid doses > 10 mg daily prednisone equivalent, are permitted in the absence of active autoimmune disease. The use of topically, ocularly, intra-articularly, intranasally and inhalationally administered corticosteroids (with minimal systemic absorption) or adrenal replacement steroid doses > 10 mg daily prednisone are permitted. A less than 3-week course of corticosteroids for prophylaxis (e.g. contrast-dye allergy) or for treatment of non-autoimmune conditions (e.g. delayed-type hypersensitivity reaction caused by a contact allergen) is permitted as is regular concomitant use of bisphosphonates and RANK-L inhibitors for prevention or reduction of skeletal-related events in patients with bone metastases if initiated prior to the first dose of study therapy.

### Statistical analysis

Analyses will be intention-to-treat (ITT), consisting of all patients who have consented and have been randomised to a treatment arm. Safety analyses will include patients who have received at least one dose of study treatment.

Time-to-event data (OS, PFS) will be analysed and presented using Kaplan-Meier curves for the ITT population. A Cox proportional hazards model will be used to calculate the HR, 95% confidence intervals and *p* value, both unadjusted and adjusted (for the randomisation stratification factor epithelioid/non-epithelioid). The adjusted Cox regression model for OS will form the primary endpoint analysis model (and for the pre-specified PD-L1 checkpoint-positive subgroups). Subgroup analyses will be undertaken in patients with negative (PD-L1 < 1%), medium (1–49%) and high (≥ 50%) PD-L1 expression, using a Cox regression model for OS and PFS adjusted for the randomisation stratification factor.

For the secondary endpoints of toxicity, ORR, QoL and treatment compliance we will compare proportions for categorical data and means/medians for continuous data using the Pearson’s *χ*^2^ test and *T* test/Mann-Whitney *U* test, respectively.

There will be no missing data imputation as the primary endpoint (and some of the secondary endpoints) is/are time-to-event data which censors at the time last seen without an event and so is included in the analysis.

### Interim analysis

The efficacy data for the PD-L1 expression-positive subgroup will be reviewed at two time points: after approximately 40% (*n* ≈ 54) and 70% (*n* ≈ 94) of patients in the PD-L1 expression-positive subgroup have been recruited and followed up for 6 months. The *p* value of < 0.001 is the Peto-Haybittle rule recommended in Pocock [17], allowing proof beyond reasonable doubt. We are planning on using this *p* value for both stopping guidelines (a symmetrical stopping boundary) as should evidence of harm or benefit arise, it needs to be sufficiently convincing to ensure that others will believe it and change their practice accordingly. In addition, a *p* value of < 0.001 is sufficiently small to preserve the *p* value of 0.04 for the final analysis with two interim analyses.

The IDMC will monitor the trial for futility. The method to use for these analyses will be planned and agreed with the IDMC and is likely to be based on the approach described by Freidlin [18]. It is anticipated that the first futility analysis (for harm) will take place after 25% of events have been observed, followed by a futility analysis after 46% of events of observed (*t*0 from Freidlin’s paper using 4% two-sided significance and 80% power), and a futility analysis after 73% of events of observed (*t*1 from Freidlin’s paper). The patients to be included in these futility analyses will be agreed with the IDMC. It is anticipated that all patients who have been randomised will be included in these analyses.

All analyses will be carried out using STATA 15.

### Health economic analysis

The economic analysis will include: (1) a ‘within-trial’ cost-effectiveness analysis, to compare the costs and health outcomes (QALYs) accrued over the follow-up period for patients in the intervention and control arms and (2) development of a cost-effectiveness model to extrapolate cost and QALY estimates over a lifetime horizon. The analyses will follow the recommended methods and ‘reference case’ recommended by NICE, including: an NHS and Personal Social Services perspective for costing; estimation of QALYs using EQ-5D data and UK value sets, and discounting of costs and QALYs at 3.5% per year. The ‘within-trial’ analysis will be pre-specified, and will take into consideration the need for multiple imputation for missing data, adjustment for baseline co-variates, inclusion of an interaction term for pre-specified subgroups (e.g. high expressers of PD-L1), and the possibility of clustering by centre. Results will be presented as a ratio – the incremental cost per QALY gained with nivolumab compared with no treatment. Non-parametric bootstrapping will be used to obtain estimates of joint uncertainty over mean costs and QALYs, which will be represented by a scatterplot on the cost-effectiveness plane, and as a cost-effectiveness acceptability curve (CEAC), showing the probability that nivolumab is cost-effective as a function of willingness to pay per QALY (the cost-effectiveness ‘threshold’). The cost-effective modelling will be conducted according to International Society for Pharmacoeconomics and Outcomes Research guidelines and probably take the form of a ‘Markov-type’ health state transition model, although we will consider whether an individual-level simulation model will add value. Probabilistic sensitivity analysis will be used to estimate how uncertainty over input parameters results in uncertainty over the model results. Modelling results will also be presented as an incremental cost-effectiveness ratio, and with a cost-effectiveness scatterplot and CEAC curve.

### Translational analysis

Samples will be sent to the central laboratory where they will be stored and analysed. The goals of the translational research will be to determine the correlation between overall survival and: (1) PD-L1 expression; (2) mutational burden (estimated by genome-wide analysis of copy number alterations) and (3) immunotranscriptomic profile. Further studies involving analysis of circulating inflammatory biomarkers, and tumour microenvironment interrogation using multiplex immunohistochemistry and transcriptome analysis, are also planned.

### Adverse event reporting

Data on adverse events will be collected at treatment and follow-up visits. The trial also has a UK regulatory compliant real-time serious adverse events reporting process to identify serious adverse reactions and suspected unexpected serious adverse reactions that could suspend/stop the trial if warranted.

### End of the trial

The end of trial is defined as when the last patient has had their last data collected.

## Discussion

Effective therapy for relapsed mesothelioma is an unmet need. Despite a significant number of clinical studies in the second-line setting, no randomised study to date has been positive.

The James Lind Alliance Priority Setting Partnership, funded by the National Institute for Health Research, has identified immunotherapy as the number-one UK research priority. To date there have been no placebo-controlled randomised trials for mesothelioma using PD-L1 or PD-1 checkpoint inhibition. Early promising signals of activity relating to both PD-L1- and PD-1-targeted treatment in mesothelioma implicate a dependency of mesothelioma on this immune checkpoint, and support the development of a randomised phase III trial to evaluate the efficacy of nivolumab.

PD-1 checkpoint inhibition has revolutionised the treatment of melanoma and is expected to become standard of care in NSCLC. It is being assessed rigorously in numerous other cancer sites, making its evaluation in mesothelioma timely in this trial. CONFIRM is the first-ever placebo-controlled, randomised phase III trial of a PD-1 immune-checkpoint inhibitor in mesothelioma (relapsed and non-relapsed). The outcome of this trial will provide evidence of the potential benefit of the use of nivolumab in the treatment of relapsed mesothelioma. If found to be clinically effective, safe and cost-effective it is likely to become the new standard of care in the UK. A potential limitation of the trial in the future could be the use of overall survival as the primary endpoint if there is any treatment crossover, as either patients on the placebo have access to immunotherapy outside of the trial or new evidence emerges that patients can be effectively re-challenged with new immunotherapy treatment combination on progression. We are currently seeking funding to add a translational component onto the trial to collect and analyse samples taken at progression to decipher mechanisms that lead to acquired resistance, and that might provide a rationale for new interventions following checkpoint-inhibitor failure. Post-progression immunotherapy (or indeed other active agents such as re-challenge pemetrexed-platinum), either by design or patient access outside of the trial, could bias the overall survival analysis. We are mitigating against this by collecting, and reporting to the Independent Data Monitoring Committee, any instances of additional treatment received by patients in each arm, and collecting detailed PFS outcome data, which could be used as an appropriate unbiased endpoint to compare the existing arms should a case be put to the funder to include an additional post-progression randomisation during the life of the trial.

Results will be disseminated to patients and clinical teams through peer-reviewed journal publications and by engaging with specialist organisations, such as Mesothelioma UK.

## Trial status

This clinical trial was registered in February 2017 (ClinicalTrials.gov, ID: NCT03063450 and ISRCTN 79814141). Recruitment opened on 28 March 2017 and is expected to be completed in March 2021). The current protocol is version 3, dated 8 February 2018. REC/MHRA-approved protocol amendments will be communicated to sites via email and updated trial documentation provided centrally via the trial website. Trial registries will be amended where relevant with explanations for these changes. Results will be published at the end of the trial in a peer-reviewed journal (authored by the members of the TMG), presented at international conferences; end of trial summaries will appear on regulatory authority databases and results fed back to recruiting sites so that any participants are able to access the results via their treating clinician.

## Additional files

Additional file 1: SPIRIT Checklist. (DOC 121 kb)

Additional file 2: Informed Consent Form. (PDF 133 kb)

## Abbreviations

ALT: Alanine aminotransferase
AST: Aspartate aminotransferase
BMS: Bristol Myers Squibb
CEAC: Cost-effectiveness acceptability curve
CNS: Central nervous system
CrCl: Creatinine clearance
CRP: C-reactive protein
CRUK: Cancer Research UK
CT: Computerised tomography
CTCAE: Common Terminology Criteria for Adverse Events
ECOG PS: Eastern Cooperative Oncology Group Performance Status
eCRF: Electronic Case Report Form
EQ-5D-5 L: European Quality of Life-5 Dimensions-5 Levels
HR: Hazard ratio
IWRS: Interactive Web Response System
LDH: Lactate dehydrogenase
MHRA: Medicines and Health Care Product Regulatory Agency
mRECIST: Modified RECIST
NCI: National Cancer Institute
NCRI: National Cancer Research Institute
NHS: National Health Service ORR Objective response rate
NICE: National Institute for Health and Clinical Excellence
OS: Overall survival
PD-1: Programmed cell death 1
PFS: Progression-free survival
PR: Partial response
QALY: Quality-adjusted life year
QoL: Quality of life
RECIST: Response Evaluation Criteria in Solid Tumours
SCTU: Southampton Clinical Trials Unit
UK: United Kingdom
ULN: Upper limit of normal
