# Supplementary material for: CONFIRM: a double-blind, placebo-controlled phase III clinical trial investigating the effect of nivolumab in patients with relapsed mesothelioma: study protocol for a randomised controlled trial
Source: Trials. 2018 Apr 18;19:233. doi: 10.1186/s13063-018-2602-y (PMC5907297; doi:10.1186/s13063-018-2602-y)
Supplement: Supplementary file 2 — Informed Consent Form. (PDF 133 kb) [file 13063_2018_2602_MOESM2_ESM.pdf]

(To be printed on local headed paper)

REC Number: 16/WM/0472
IRAS ID: 211270

CONFIRM CONSENT FORM

CONFIRM: CheckpOiNt blockade For Inhibition of Relapsed Mesothelioma: A phase III trial to evaluate the efficacy of Nivolumab in relapsed mesothelioma

Patient Identification Number for this trial: | | | | | | | |

Name of Researcher:

	Please initial box
1. I confirm that I have read and understand the information sheet dated................... (version...........) for the above study. I have had the opportunity to consider the information, ask questions and have had these answered satisfactorily.	INITIAL
2. I understand that my participation is voluntary and that I am free to withdraw at any time without giving any reason, without my medical care or legal rights being affected.	INITIAL
3. I consent to the storage of personal information (including electronic) for the purposes of this study. I understand that any information that could identify me will be kept strictly confidential and that no personal information will be included in the study report or other publication.	INITIAL
4. I understand that relevant sections of my medical notes and data collected during the study, may be looked at by individuals from Southampton Clinical Trials Unit, Study Sponsor organization, regulatory authorities or from the NHS Trust, where it is relevant to my taking part in this research and understand that the anonymised data could be transferred both within and outside the EEA (for example, the USA).	INITIAL
5. I understand that the information collected about me will be used to support other research in the future, and may be shared anonymously with other researchers.	INITIAL
6. I agree for my details to be registered with the National Health Service Digital or equivalent for which my name and NHS number must be used in order for my health status to be followed up.	INITIAL
7. I consent to give research blood sample(s) for use in laboratory research studies including genetic analysis: Blood DNA (extracted from blood)	INITIAL
8. I consent to the study doctor requesting tissue samples from my previous tumour biopsy and diagnostic biopsy tissue (where available). If this sample is not available, I agree to provide a re-biopsy.	INITIAL

I understand that these samples will be stored and may be used for future research purposes, which may include genetic testing.	
9. I agree to my anonymised data being used in future ethically approved research.	INITIAL
10. I agree to my GP being informed of my participation in the study.	INITIAL
11. I agree to use effective contraception as detailed in the patient information sheet.	INITIAL
12. I understand that I shall not benefit financially even if future research leads to the development of new treatments or medical tests.	INITIAL

13. **WOMEN OF CHILD BEARING POTENTIAL:** I understand that I will need to take a pregnancy test at screening and at routine monthly intervals as detailed in the patient information sheet.	INITIAL

	Yes	No
14. **OPTIONAL:** I agree to provide a second biopsy for research purposes if my cancer progresses	INITIAL	INITIAL

**15. I agree to take part in the CONFIRM study.**	INITIAL

Name of Patient	Signature	Date
Name of researcher taking consent	Signature	Date

When completed: 1 for participant; 1 for researcher site file; 1 (original) to be kept in medical notes.
